# Efficacy and Safety of Tirzepatide and Semaglutide for Obesity Management: A Real-World Comparison

**DOI:** 10.7759/cureus.98858

**Published:** 2025-12-09

**Authors:** Kishore Kumar Shil, Ananda D Hira, Sudipta Bakchi, Susanta K Paul, Mahmud Hossain, Debasish K Ghosh

**Affiliations:** 1 Endocrinology, Khulna Medical College, Khulna, BGD; 2 Pathology, Khulna Medical College Hospital, Khulna, BGD; 3 Cardiology, Khulna Medical College Hospital, Khulna, BGD; 4 Pulmonology, Khulna Medical College Hospital, Khulna, BGD; 5 Rheumatology, Khulna Medical College Hospital, Khulna, BGD; 6 Endocrinology, Khulna Medical College Hospital, Khulna, BGD

**Keywords:** community obesity, lifestyle intervention, semaglutide, “tirzepatide”, weight loss and obesity

## Abstract

Background

Incretin-based therapies have emerged as effective strategies for obesity and type 2 diabetes mellitus (T2DM) management. Semaglutide and tirzepatide are among the most promising agents. Real-world data in Bangladesh on their efficacy, lifestyle interactions, and safety profiles remain limited. The objective of the study is to evaluate the associations of tirzepatide and semaglutide with weight reduction and safety in adults with obesity, and to assess the influence of lifestyle factors on treatment outcomes.

Methods

This retrospective observational study analyzed the electronic medical records of 100 adults with obesity from three tertiary centers in Bangladesh (58 patients treated with tirzepatide, 42 patients treated with semaglutide) over one year. Participants had completed at least three months of therapy. Demographics, clinical parameters, comorbidities, lifestyle adherence, weight change, and adverse events were collected using a structured questionnaire.

Results

A total of 100 participants (58 tirzepatide, 42 semaglutide; median age 27 years) were included, with 87 (87%) being female. Baseline BMI was higher in the tirzepatide group (36.6±4.77 vs. 32.3±3.55 kg/m²; p<0.001). Both drugs produced significant weight loss, with tirzepatide achieving greater reductions than semaglutide (8.53±4.10 vs. 6.85±3.38 kg; p=0.033). Clinically meaningful weight loss (≥5%) was observed in 89 (89%) of participants, and ≥10% loss in 33 (33%), with higher proportions in the tirzepatide group. Lifestyle interventions further enhanced weight reduction, most notably with tirzepatide. Gastrointestinal adverse events were the most common, occurring more frequently with tirzepatide, while serious events were rare. Overall, tirzepatide was associated with greater weight reduction, especially when combined with lifestyle modification.

Conclusion

Tirzepatide and semaglutide were both associated with weight reduction in obesity, with greater reductions observed with tirzepatide, particularly when combined with lifestyle interventions, underscoring the importance of combining behavioral and pharmacological strategies.

## Introduction

The global prevalence of obesity has escalated into a public health crisis, with over one billion individuals affected worldwide. The World Obesity Atlas 2023 predicts that, without effective intervention, more than half of the global adult population will be overweight or obese by 2050 [1]. In South Asia, the situation is equally concerning. A study published in Scientific Reports indicates that the pooled prevalence of obesity in South Asia is 6.6%, with Bangladesh reporting a higher rate of 8.9% [2]. This rising trend is attributed to urbanization, sedentary lifestyles, and dietary shifts towards high-calorie foods. In Bangladesh, while the national obesity prevalence is lower than the regional average, urban areas exhibit higher rates, with childhood obesity reaching 10.89%, particularly among urban children [3]. The adverse impacts of obesity are profound, leading to an increased burden of non-communicable diseases such as type 2 diabetes mellitus (T2DM), hypertension, obstructive sleep apnea, and cardiovascular diseases. It also has some adverse effects on mental issues. Moreover, obesity contributes to economic strain due to increased healthcare costs and reduced productivity. Addressing this issue requires a multifaceted approach, including public health initiatives, lifestyle modifications, and healthcare system strengthening and integrated obesity management.

In the era of anti-obesity treatments, semaglutide and tirzepatide have gathered significant attention due to their efficacy and safety profiles in weight management. Both medications act as glucagon-like peptide-1 (GLP-1) receptor agonists, although tirzepatide also targets the glucose-dependent insulinotropic polypeptide (GIP) receptor, thus enhancing its therapeutic potential. Semaglutide has been widely studied for its efficacy in achieving significant weight loss, showing marked results in both clinical trials and real-world settings [4,5]. On the other hand, tirzepatide, through its dual action on GLP-1 and GIP receptors, presents a novel therapeutic mechanism, potentially offering superior weight loss advantages over semaglutide when combined with lifestyle modifications [6]. The comparative efficacy of tirzepatide over semaglutide in achieving weight loss has been demonstrated, with studies indicating greater weight reduction results with tirzepatide [7].

Both medications have shown promising outcomes in clinical settings, with patients achieving significant weight loss by stimulating the satiety centre, delayed gastric emptying, and modulating other metabolic pathways. Tirzepatide's dual action enables more pronounced effects on body weight reduction by not only enhancing glycemic control but also by impacting metabolic pathways more comprehensively compared to semaglutide [8].

Compounded/non-comparable medications are custom-made formulations combining active ingredients to provide tailored therapeutic effects. For example, a compounded tirzepatide-semaglutide formulation can exploit dual-receptor activity to enhance metabolic benefits and weight loss. While safety concerns exist, such compounded versions are valuable in countries like Bangladesh, where cost and access to original drugs are limited, offering a more affordable and accessible option [9].

Both drugs, however, share similar side effect profiles, primarily gastrointestinal in nature, including nausea, vomiting, and diarrhea [10].

The increasing prevalence of obesity worldwide underscores the need for effective therapeutic options. The superior efficacy of compounded medications, like tirzepatide, in managing obesity presents a significant advancement in pharmacotherapy, offering healthcare professionals new tools in combating obesity-related health complications in developing countries like Bangladesh, where original molecules are unavailable and also costly [11]. Studying these drugs in the real world further could illuminate new pathways in personalized medicine, enhancing treatment outcomes on a larger scale [12].

## Materials and methods

This retrospective observational study analyzed electronic medical records from tertiary healthcare facilities across three regions of Bangladesh over one year (from May 7, 2024, to May 8, 2025). A total of 100 participants were included, with 58 participants receiving tirzepatide and 42 participants receiving semaglutide. The study design was observational and descriptive; therefore, no formal sample size calculation was performed. All eligible records within the study period were included to maximize the available data for real-world insights into the efficacy and safety of tirzepatide and semaglutide in routine clinical practice. Eligible participants were adults (≥18 years) of both sexes who met the BMI criteria for obesity, had received the prescribed anti-obesity drug for at least three months, and possessed complete baseline and follow-up data, regardless of glycemic status. The study protocol was reviewed and approved by the Ethical Review Committee of Khulna Medical College Hospital.

Data were systematically extracted using a standardized data extraction form developed by the research team. The form captured demographic information (age, sex, residence, occupation, monthly income), clinical parameters (height, weight, BMI, blood pressure), comorbidities, medication history, lifestyle adherence (diet and physical activity as recorded by clinicians), treatment details (drug type, dose, frequency, and adverse effects), and outcomes (weight and BMI change at three months). The extraction form was pilot-tested on a subset of records to ensure clarity, completeness, and consistency, and culturally refined for local relevance.

Participants underwent clinical evaluation through history, physical examination, and relevant investigations to diagnose obesity, its complications, and comorbidities at baseline and follow-up. Data collection included demographic details (residence, age, sex, occupation, monthly income), medication history, diabetes status and treatment, as well as clinical parameters such as height, weight, BMI, and blood pressure. Concomitant medications, including treatments for diabetes, hypertension, hypothyroidism, and polycystic ovary syndrome (PCOS), were recorded from medical records to account for potential influences on efficacy and safety outcomes. Dietary and lifestyle recommendations were individualized according to each patient's comorbidities, cultural preferences, and affordability. All recommendations and adherence assessments were obtained from routine clinical documentation, with no additional interventions by the study team. Biochemical data were obtained from results documented in accredited laboratories during routine clinical care at baseline and follow-up visits. Height was measured with a stadiometer, weight with a calibrated balance on a hard surface, and blood pressure using a standard analog (manual) sphygmomanometer, consisting of an inflatable cuff and an aneroid manometer, following established clinical guidelines. Blood pressure categories were defined as normal (<120/80 mmHg), elevated (120-129/<80 mmHg), and hypertensive (≥130/80 mmHg) [13].

BMI was categorized according to the World Health Organization (WHO) adult obesity classification for Asian populations: underweight (<18.5 kg/m²); normal weight (18.5-22.9 kg/m²); overweight (23-24.9 kg/m²); and obese (≥25 kg/m²). Obesity was further categorized into Class 1 (25-29.9 kg/m²), Class 2 (30-34.9 kg/m²), and Class 3 (≥35 kg/m²) [14].

Dietary adherence was evaluated according to individualized recommendations tailored to comorbidities, cultural preferences, and affordability. A caloric deficit of 500-750 kcal/day was advised, aiming for approximately 0.5-1 kg weight loss per week. Standard guidance included three main meals and two to three snacks daily, appropriate portion control, and adjustments for specific conditions (e.g., type 1 diabetes, eating disorders, inflammatory bowel disease, non-alcoholic fatty liver disease, celiac disease, or neurodiversity-related restrictions). Adherence was categorized as: (1) full adherence - daily compliance with dietary guidelines, including portion control and target calorie intake; (2) non-adherence - rare or no compliance with portion control and target calorie intake.

Exercise adherence was assessed based on self-reported physical activity and classified as: (1) adherent - engaging in 45-60 minutes of moderate-intensity activity daily; (2) non-adherent - no regular physical activity [15].

At baseline and during routine follow-up visits, participants received counseling on diet, physical activity, behavioral modification, and medication adherence as documented in their medical records. For patients treated with tirzepatide, the documented regimen included initiation at 2.5 mg weekly for the first month, followed by an increase to 5 mg weekly for the next two months. In the semaglutide group, records indicated therapy began at 0.25 mg weekly for the first month and increased to 0.5 mg weekly for the subsequent two months. Both groups received locally available biologic brands as prescribed by their treating clinicians. Participants were reviewed after one month for severe adverse effects and again at three months. At each visit, lifestyle changes, anthropometric measurements, comorbidities, complications, drug adherence, and side effects were assessed. Dose adjustments and treatment continuation were determined in discussion with participants. Prescription records were used to document baseline characteristics, medication dose, number of injections between visits, weight, side effects, dosing decisions, and adherence. Weight change was calculated as the difference between baseline and follow-up weights, expressed as a percentage of baseline weight. Only participants who completed three months of follow-up with complete data were included in the analysis.

Data were entered, cleaned, and analyzed using SPSS version 25.0 (IBM, Inc., Armonk, US). Quantitative variables were summarized as mean ± standard deviation (SD) or median with interquartile range (IQR), while categorical variables were reported as frequencies and percentages. Normality was assessed using the Shapiro-Wilk test, which indicated that all variables were normally distributed except age. Associations were examined using the chi-square or Fisher's exact test for categorical variables, and the unpaired t-test and Mann-Whitney U-test for continuous variables, as appropriate. A p-value <0.05 was considered statistically significant.

This retrospective observational study was conducted in accordance with the ethical principles outlined in the Declaration of Helsinki. The study protocol was reviewed and approved by the Ethical Review Committee of Khulna Medical College Hospital, Bangladesh (Ref: KMC/ERC/03; dated March 5, 2025). Written informed consent was obtained at the time of treatment for the use of patients' medical records for research purposes, as per hospital policy and ethical guidelines. For participants whose prior consent for research use was not documented, the study used only de-identified data to protect privacy, and the Institutional Ethical Committee approved a waiver of additional consent for retrospective data analysis. All patient information was anonymized and handled with strict confidentiality to ensure privacy and data protection.

## Results

Baseline characteristics

A total of 100 participants were enrolled, with 58 receiving tirzepatide and 42 receiving semaglutide. Participants' median age was 27 years (IQR: 22-35), and there was no discernible age group difference (p=0.12). Additionally, there was no discernible difference between the groups in terms of residency, monthly income, sex, diet, and exercise. The majority of the participants, 61 (61%), were aged 18-30 years, and 87 (87%) of the participants were female. Most participants, 71 (71%), reported a monthly household income of Tk. 21,000-40,000, and 55 (55%) of the population resided in urban areas. Blood pressure distribution differed marginally between groups (p=0.051; Table 1).

**Table 1:** Baseline characteristics of the participants Median (IQR) for skewed data. * Chi-square was calculated for age, and the Mann-Whitney U-test for categorical variables.

Characteristics	All participants	Tirzepatide group (n=58)	Semaglutide group (n=42)	p-value	U-value/chi-square value
†Age (years), median (IQR)	27 (22-35)	28.00 (23.75-36.0)	24.50 (19.75-35.25)	0.12	†999.00
Age category, n (%)					
18-30	61 (61%)	34 (58.62%)	27 (64.28%)	0.664	0.82
30-40	21 (21%)	14 (24.13%)	7 (16.6650		
≥ 41	18 (18%)	10 (17.24%)	8 (19.04%)		
Sex, n (%)					
Male	13 (13%)	7 (12.06%)	6 (14.28%)	0.665	0.817
						Female	87 (87%)	51 (87.93%)	36 (85.71%)
Income category (tk.), n (%)					
<20000	6 (6%)	3 (5.17%)	3 (7.14%)	0.88	0.256
21000-40000	71 (71%)	43 (74.13%)	28 (66.66%)		
≥41000	23 (23%)	12 (20.68%)	11 (26.19%)		
Residence, n (%)					
Rural	45 (45%)	23 (39.65%)	22 (52.38%)	0.207	1.59
Urban	55 (55%)	35 (60.34%)	20 (47.61%)		
Blood pressure, n (%)					
Normal	60 (60%)	29 (50%)	31 (73.80%)	0.051	5.94
Elevated	23 (23%)	16 (27.58%)	7 (16.66%)		
High	17 (17%)	13 (22.41%)	4 (9.52%)		
Diet, n (%)					
No	49 (49%)	24 (41.37%	25 (59.51%)	0.07	3.2
Yes	51 (51%)	34 (58.62%)	17 (40.47%)		
Exercise, n (%)					
No	70 (70%)	38 (65.51%)	32 (76.18%)	0.25	1.32
Yes	30 (30%)	20 (34.48%)	10 (23.80%)		
Both diet and exercise, n (%)					
No	76 (76%)	42 (72.41%)	34 (80.95%)	0.32	0.97
Yes	24 (24%)	16 (27.58%)	8 (19.04%)		
Antidiabetic medication, n (%)					
No	80 (80%)	47 (81.03%)	33 (78.57%)	0.76	0.09
Yes	20 (20%)	11 (18.96%)	9 (21.42%)		

Among all participants, in 27 (27%) patients, PCOS was the most common comorbidity, followed by diabetes mellitus in 18 (18%) patients and hypothyroidism in 17 (17%). Most participants were students, 47 (47%), and 42 (42%) reported no regular medication use, while 22 (22%) were on PCOS treatment and 10 (10%) used levothyroxine (Table 2).

**Table 2:** Baseline distribution of participants by comorbidities, occupation, and medication history * Other: Graves' disease, prediabetes, MASLD, subclinical hypothyroidism; ** carbimazole, antihypertensive DM - diabetes mellitus; PCOS - polycystic ovary syndrome; MASLD - metabolic dysfunction–associated steatotic liver disease

Characteristics	All participants
Types of comorbidities, n (%)	
DM	18 (18%)
Hypothyroidism	17 (17%)
PCOS	27 (27%)
Other*	08 (08%)
No	35 (35%)
Occupation, n (%)	
Student	47 (47%)
Housewife	33 (33%)
Service holder	12 (12%)
Businessman	8 (8%)
Medication, n (%)	
For PCOS	22 (22%)
Levothyroxine	10 (10%)
No	42 (42%)
Other**	5 (5%)

Anthropometric outcomes

The mean baseline BMI was 34.8±4.78 kg/m², significantly higher in the tirzepatide group compared to semaglutide (36.6±4.77 vs. 32.3±3.55; p<0.001). Obesity classification also differed significantly (p<0.001): Class 1 obesity was more common among semaglutide users (21.4% vs. 3.4%), while Class 3 obesity was more prevalent among tirzepatide users (62.1% vs. 23.8%). Both agents resulted in weight reduction, with a mean overall loss of 7.83±3.89 kg. Tirzepatide achieved a significantly greater weight reduction compared to semaglutide (8.53±4.10 vs. 6.85±3.38 kg; p=0.033; mean difference of 1.7 kg; Table 3).

**Table 3:** Changes in body weight * t-statistic was calculated for BMI and weight change; chi-square value for BMI category

Variables	All participants (n=100)	Tirzepatide group (n=58)	Semaglutide group (n=42)	p-value	t-statistic/chi-square value*
BMI (mean±SD, 95% CI)	34.80±4.78 (33.87-35.73)	36.60±4.77 (35.46-37.74)	32.32±3.55 (31.16-33.48)	<0.001	-5.13
Weight change (kg, mean±SD, 95% CI)	7.83±3.89 (7.16-8.50)	8.53±4.10 (7.52-9.54)	6.85±3.38 (5.87-7.83)	0.033	-2.16
BMI category					
Class 1 obesity	11 (11%)	2 (3.44%)	9 (21.42%)	<0.001	17.24
Class 2 obesity	43 (43%)	20 (43.48%)	23 (54.76%)		
Class 3 obesity	46 (46%)	36 (62.06%)	10 (23.80%)		

Proportion of clinically meaningful weight loss

**Figure 1:**
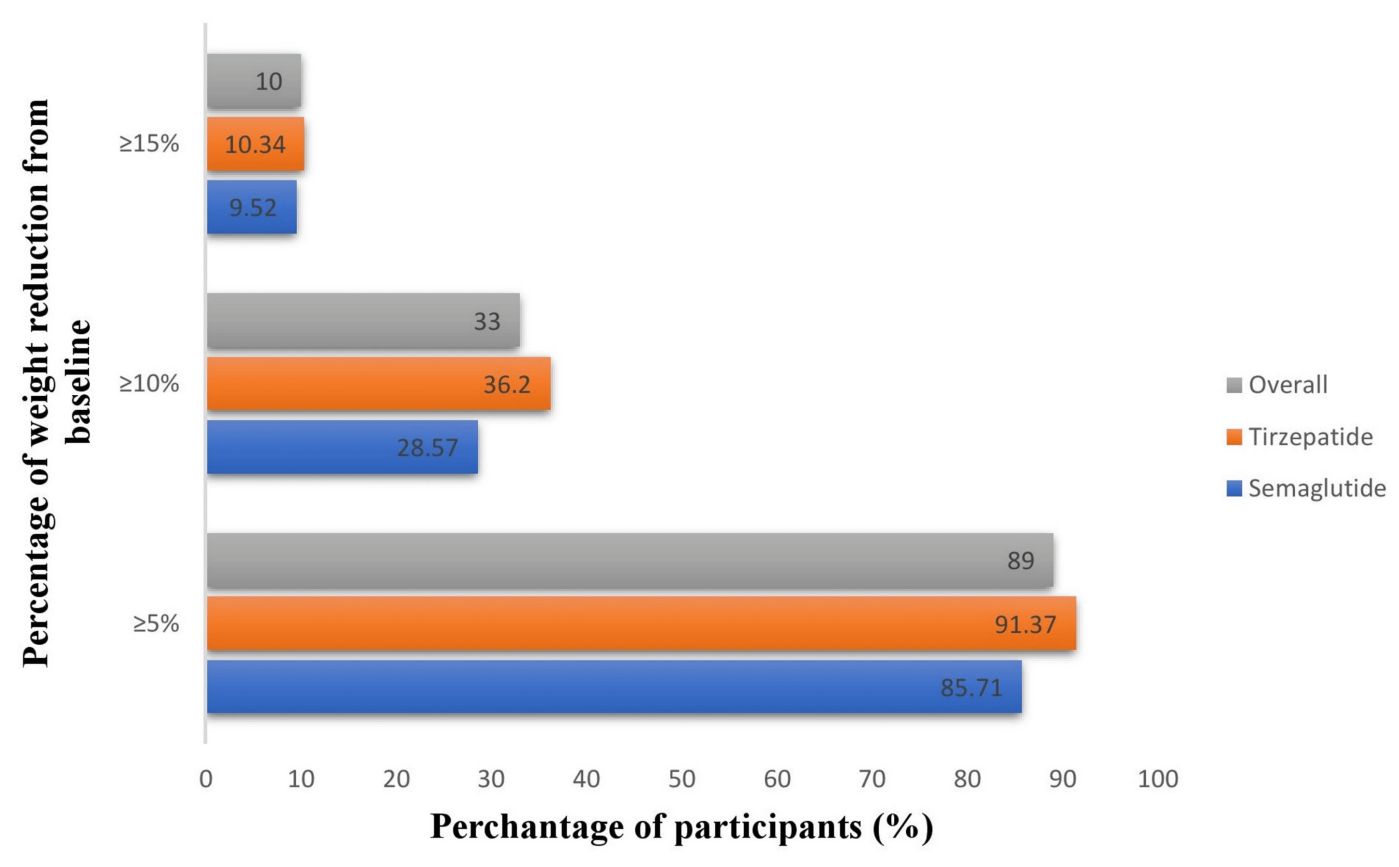
Changes in percentage of body weight

At least 5% weight reduction was achieved by 89 (89%) of participants, with a higher proportion in the tirzepatide group (91.4% vs. 85.7%). Similarly, ≥10% weight loss occurred in 33 (33%) patients (36.2% in the tirzepatide group vs. 28.6% in the semaglutide group). A ≥15% reduction was less common, observed in 10 (10%) overall, with comparable rates between tirzepatide (10.3%) and semaglutide (9.5%; Figure 1).

Lifestyle factors and weight reduction

Lifestyle interventions significantly influenced outcomes (Table 4). Participants with dietary modification lost more weight than those without (9.76±4.02 vs. 5.81±2.49 kg; p<0.001; mean difference of 3.95 kg). Within groups, the effect of diet was stronger for tirzepatide (10.23±4.22 vs. 6.12±2.41 kg) than for semaglutide (8.82±3.53 vs. 5.52±2.58 kg). Exercise also enhanced weight loss (11.26±4.25 vs. 6.35±2.61 kg; p<0.001). The benefit was observed in both tirzepatide (11.60±4.51 vs. 6.92±2.78 kg) and semaglutide groups (10.60±3.38 vs. 5.68±2.24 kg). The combination of diet and exercise produced the greatest effect (12.16±4.05 vs. 6.46±2.65 kg; p<0.001). This effect was most pronounced among tirzepatide users (12.81±4.11 vs. 6.90±2.71 kg) compared to semaglutide users (10.87±3.83 vs. 5.91±2.51 kg). Antidiabetic medication use was not significantly associated with weight reduction.

**Table 4:** Comparison of effects of different variables on weight change Mean±SD for normally distributed data. Significance level was measured by an unpaired t-test.

Variables	All participants (n=100)			Tirzepatide group (n=58)			Semaglutide group (n=42)		
	Mean±SD	p-value	t-statistic	Mean±SD	p-value	t-statistic	Mean±SD	p-value	t-statistic
Diet									
No	5.81±2.49	<0.001	-5.91	6.12±2.41	<0.001	-4.69	5.52±2.58	0.001	-3.49
Yes	9.76±4.02			10.23±4.22			8.82±3.53		
Exercise									
No	6.35±2.61	<0.001	-5.87	6.92±2.78	0.005	-4.23	5.68±2.24	<0.001	-5.06
Yes	11.26±4.25			11.60±4.51			10.60±3.38		
Both diet and exercise									
No	6.46+2.65	<0.001	-6.47	6.90+2.71	<0.001	-5.31	5.91+2.51	<0.001	-3.48
Yes	12.16±4.05			12.81±4.11			10.87±3.83		
Antidiabetic medication									
No	8.12±3.99	0.13	1.52	9.04±4.14	0.05	1.99	6.81±3.42	0.88	-0.14
Yes	6.65±3.28			6.36±3.26			7.00±3.46		

Adverse effects

Gastrointestinal adverse events were the most common (Table 5). Anorexia, 62 (62%), acidity, 47 (47%), nausea, 35 (35%), vomiting, 40 (40%), and diarrhea, 32 (32%) were reported, with higher frequencies of nausea (43.1% vs. 23.8%), vomiting (48.3% vs. 28.6%), and diarrhea (39.7% vs. 21.4%) in the tirzepatide group. Constipation (2%; 2%), dizziness (5%; 5%), abdominal pain (6%; 6%), and headache (10%; 10%) were less frequent. Vertigo was reported by 21 (21%) participants, more common in tirzepatide users (25.9% vs. 14.3%). Injection-site reactions (1, 1%), a feverish feeling/malaise (2, 2%), and pancreatitis (1, 1%) were rare and occurred only in tirzepatide users. No cases of cholecystitis, hypoglycemia, or death were observed.

**Table 5:** Spectrum of side-effects * Other: postural dizziness, dry mouth, hyperactivity

Side effect	All participants (n=100)	Tirzepatide group (n=58)	Semaglutide group (n=42)
Heart burn	47 (47%)	27 (46.55%)	20 (47.61%)
Anorexia	62 (62%)	37 (63.79%)	25 (59.52%)
Nausea	35 (35%)	25 (43.10%)	10 (23.80%)
Vomiting	40 (40%)	28 (48.27%)	12 (28.57%)
Diarrhoea	32 (32%)	23 (39.65%)	9 (21.42%)
Constipation	2 (2%)	1 (1.72%)	1 (2.38%)
Dizziness	5 (5%)	3 (5.17%)	2 (4.76%)
Weakness	42 (42%)	24 (41.37%)	18 (42.85%)
Abdominal pain	6 (6%)	5 (8.62%)	1 (2.38%)
Headache	10 (10%)	8 (13.79%)	2 (4.76%)
Injection site reaction	1 (1%)	1 (1.72%)	0 (0%)
Feverish feeling/ malaise	2 (2%)	2 (3.44%)	0 (0%)
Vertigo	21 (21%)	15 (25.86%)	6 (14.28%)
Pancreatitis	1 (1%)	1 (1.72%)	0 (0%)
Cholecystitis	0 (0%)	0 (0%)	0 (0%)
Hypoglycemia	0 (0%)	0 (0%)	00 (0%)
Other*	3 (3%)	2 (3.44%)	1 (2.38%)
No	2 (2%)	1 (1.72%)	1 (2.38%)
Death	0 (0%)	0 (0%)	0 (0%)

Overall, both tirzepatide and semaglutide produced clinically meaningful weight loss, particularly when combined with lifestyle modification. Tirzepatide was associated with greater reductions in weight and BMI.

## Discussion

In this retrospective observational analysis of 100 participants, tirzepatide was associated with a greater magnitude of short-term weight reduction than semaglutide. Participants who adhered to lifestyle interventions, including diet and exercise, achieved enhanced weight loss, highlighting the potential additive effect of lifestyle measures. Gastrointestinal adverse events, particularly nausea, vomiting, and diarrhea, were slightly more common in the tirzepatide group. However, differences in baseline BMI, obesity class, and other participant characteristics likely influenced outcomes, and no adjustment for these confounders was performed. Therefore, the observed differences should be interpreted as associations rather than evidence of comparative efficacy.

Our observations are broadly consistent with results from clinical trials and meta-analyses, which indicate that tirzepatide can achieve greater weight reduction than semaglutide, particularly at higher doses [16-19]. In these studies, the magnitude of difference depends on baseline characteristics, dosing, and follow-up duration. For example, a study of tirzepatide versus semaglutide once weekly as add-on therapy to metformin in participants with type 2 diabetes (SURPASS-2) reported 10-12 kg weight loss with tirzepatide 10-15 mg versus ~6 kg with semaglutide 1 mg [18]. Similarly, real-world analyses suggest tirzepatide users are more likely to achieve ≥5%, ≥10%, and ≥15% weight loss at 3-12 months compared to semaglutide users [16,20]. In our smaller cohort, a higher proportion of tirzepatide-treated participants achieved ≥5% and ≥10% weight loss, although this finding may reflect baseline BMI differences and the retrospective nature of the study.

The adverse event profile observed in our cohort mirrors prior reports, with gastrointestinal symptoms predominating and more frequently reported at higher tirzepatide doses [17,21]. Lifestyle adherence was associated with improved outcomes, underscoring the importance of combined diet and exercise interventions alongside pharmacotherapy. This aligns with prior studies showing that GLP-1 receptor agonists paired with lifestyle modification more effectively improve weight and metabolic outcomes than either approach alone [22].

Mechanistically, tirzepatide's dual GIP and GLP-1 receptor agonism may enhance metabolic effects relative to semaglutide, which targets only GLP-1 receptors. This dual activity may contribute to the observed greater weight reduction and possibly the slightly higher rate of gastrointestinal adverse events. Concomitant medications, including those for diabetes, hypertension, hypothyroidism, and PCOS, were recorded from medical records, with no clinically significant interactions noted.

Although most clinical evidence pertains to originator products, our study demonstrates that locally available non-comparable biologics also achieved meaningful weight reduction over a short three-month period, even at lower doses. While these findings provide initial real-world insights into the Bangladeshi population, larger, prospective studies with adjusted analyses and longer follow-up are needed to more reliably assess efficacy, safety, and optimal integration of pharmacotherapy with lifestyle interventions in obesity management.

Key strengths of this study include the use of real-world multicenter data from Bangladesh, standardized data collection, and comprehensive evaluation of clinical, biochemical, and lifestyle parameters. The comparison of tirzepatide and semaglutide, tirzepatide (2.5-5 mg weekly) and semaglutide (0.25-0.5 mg weekly) over a three-month follow-up period, along with lifestyle adherence, offers practical insights relevant to local clinical practice, particularly regarding non-comparable biologics. The study is limited by its retrospective design, small sample size, short follow-up, reliance on self-reported lifestyle data, and unaccounted confounders, including baseline group differences, comorbidities, discontinuation rates, and dose adjustments. Because only unadjusted comparisons were performed, residual confounding is likely. The use of non-comparable biologics further restricts generalizability, and the findings should therefore be interpreted with caution.

Overall, this study underscores tirzepatide's superior short-term weight loss efficacy, particularly when combined with lifestyle interventions, reinforcing the importance of integrated pharmacologic and behavioral strategies. In resource-limited settings such as Bangladesh, rigorous evaluation of non-comparable biologics is essential to ensure safety and efficacy. Future investigations should aim for larger, longer-term trials to assess sustained weight outcomes, safety, and adherence strategies for optimizing obesity management in real-world practice.

## Conclusions

In this real-world study, the tirzepatide group showed a greater magnitude of short-term weight reduction than the semaglutide group; however, this observation should be interpreted cautiously due to baseline BMI differences and the retrospective design. Both medications were generally well tolerated, with gastrointestinal symptoms being the most frequently reported side effects, particularly at higher tirzepatide doses. Although the use of locally available non-reference biologics in Bangladesh provides practical insights into real-world practice, careful monitoring and patient education remain essential. Future studies with larger sample sizes, longer follow-up, and adjusted analyses are needed to confirm long-term efficacy, safety, and optimal integration of pharmacotherapy with lifestyle interventions in obesity management.
